# General Supervised Learning as Change Propagation with Delta Lenses

**DOI:** 10.1007/978-3-030-45231-5_10

**Published:** 2020-04-17

**Authors:** Zinovy Diskin

**Affiliations:** 8 ENS/CNRS, Université Paris-Saclay, Cachan, France; 9 University of Duisburg-Essen, Duisburg, Germany; McMaster University, Hamilton, Canada

## Abstract

Delta lenses are an established mathematical framework for modelling and designing bidirectional model transformations (Bx). Following the recent observations by Fong et al, the paper extends the delta lens framework with a a new ingredient: learning over a parameterized space of model transformations seen as functors. We will define a notion of an asymmetric learning delta lens with amendment (ala-lens), and show how ala-lenses can be organized into a symmetric monoidal (sm) category. We also show that sequential and parallel composition of well-behaved (wb) ala-lenses are also wb so that wb ala-lenses constitute a full sm-subcategory of ala-lenses.

### 1 Introduction

The goal of the paper is to develop a formal model of *supervised learning* in a very general context of *bidirectional model transformation* or *Bx*, i.e., synchronization of two arbitrary complex structures (called *models*) related by a transformation.[1] Rather than learning parameterized functions between Euclidean spaces as is typical for machine learning (ML), we will consider learning mappings between model spaces and formalize them as parameterized functors between categories, $f\colon P\times\mathbf{A} \to \mathbf{B}$, with $P$ being a parameter space. The basic ML-notion of a *training pair* $(A, B') \in \mathbf{A}_0 \times \mathbf{B}_0$ will be considered as an inconsistency between models caused by a change (*delta*) $v\colon B \to B'$ of the target model $B = f(p, A)$, $p \in P$, that was first consistent with $A$ w.r.t. the transformation (functor) $f(p, \_)$. An inconsistency is repaired by an appropriate change of the source structure, $u\colon A \to A'$, changing the parameter $p$ to $p'$, and an *amendment* of the target structure $v^@\colon B' \to B^@$ so that $f(p', A') = B^@$ is a consistent state of the parameterized two-model system.

The setting above without parameterization and learning (i.e., $p' = p$ always holds), and without amendment ($v^@ = \mathsf{id}_{B'}$ always holds), is well known in the Bx literature under the name of *delta lenses*— mathematical structures, in

[1] Term *Bx* refers to a wide area including file synchronization, data exchange in databases, and model synchronization in Model-Driven software Engineering (MDE), see [7] for a survey. In the present paper, Bx will mainly refer to Bx in the MDE context.

which consistency restoration via change propagation is modelled by functorial-like algebraic operations over categories [12,6]. There are several types of delta lenses tailored for modelling different synchronization tasks and scenarios, particularly, symmetric and asymmetric. In the paper, we only consider asymmetric delta lenses and will often omit explicit mentioning these attributes. Despite their extra-generality, (delta) lenses have been proved useful in the design and implementation of practical model synchronization systems with triple graph grammars (TGG) [5,2]; enriching lenses with amendment is a recent extension of the framework motivated and formalized in [11]. A major advantage of the lens framework for synchronization is its compositionality: a lens satisfying several equational laws specifying basic synchronization requirements is called *well-behaved (wb)*, and basic lens theorems state that sequential and parallel composition of wb lenses is again wb. In practical applications, it allows the designer of a complex synchronizer to avoid integration testing: if elementary synchronizers are tested and proved to be wb, their composition is automatically wb too.

The present paper makes the following contributions to the delta lens framework for Bx. a) We motivate model synchronization enriched with learning and, moreover, with *categorical* learning, in which the parameter space is a category, and introduce the notion of a *wb asymmetric learning (delta) lens* with *amendment* (a *wb ala-lens*) (this is the content of Sect. 3). b) We prove compositionality of wb ala-lenses and show how their universe can be organized into a symmetric monoidal (sm) category (Theorems 1-3 in Sect. 4). All proofs (rather straightforward but notationally laborious) can be found in the long version of the paper [9]. One more compositional result is c) a definition of a *compositional bidirectional transformation language* (Def. 6) that formalizes an important requirement to model synchronization tools, which (surprisingly) is missing from the Bx literature. Background Sect. 2 provides a simple example demonstrating main concepts of Bx and delta lenses in the MDE context. Section 5 briefly surveys related work, and Sect. 6 concludes.

*Notation.* Given a category $\mathbf{A}$, its objects are denoted by capital letters $A$, $A'$, etc. to recall that in MDE applications, objects are complex structures, which themselves have elements $a, a', ....$; the collection of all objects of category $\mathbf{A}$ is denoted by $\mathbf{A}_0$. An arrow with domain $A \in \mathbf{A}_0$ is written as $u\colon A \to \_$ or $u \in \mathbf{A}(A, \_)$; we also write $\mathsf{dom}(u) = A$ (and sometimes $u^{\mathsf{dom}} = A$ to shorten formulas). Similarly, formula $u\colon \_ \to A'$ denotes an arrow with codomain $u.\mathsf{cod} = A'$. Given a functor $f\colon \mathbf{A} \to \mathbf{B}$, its object function is denoted by $f_0\colon \mathbf{A}_0 \to \mathbf{B}_0$.

A subcategory $\mathbf{B} \subset \mathbf{A}$ is called *wide* if it has the same objects. All categories we consider in the paper are small.

### 2 Background: Update propagation and delta lenses

Although Bx ideas work well only in domains conforming to the slogan *any implementation satisfying the specification is good enough* such as code generation (see [10] for discussion), and have limited applications in databases (only so called updatable views can be treated in the Bx-way), we will employ a simple database example: it allows demonstrating the core ideas without any special domain knowledge required by typical Bx-amenable areas. The presentation will be semi-formal as our goal is to motivate the delta lens formalism that abstracts the details away rather than formalize the example as such.

#### 2.1 Why deltas.

Bx-lenses first appeared in the work on file synchronization, and if we have two sets of strings, say, $B = \{\text{John}, \text{Mary}\}$ and $B' = \{\text{Jon}, \text{Mary}\}$, we can readily see the difference: John $\neq$ Jon but Mary $=$ Mary. We thus have a structure in-between $B$ and $B'$ (which maybe rather complex if $B$ and $B'$ are big files), but this structure can be recovered by string matching and thus updates can be identified with pairs. The situation dramatically changes if $B$ and $B'$ are object structures, e.g., $B = \{o_1, o_2\}$ with $\mathsf{Name}(o_1) = \text{John}$, $\mathsf{Name}(o_2) = \text{Mary}$ and similarly $B' = \{o'_1, o'_2\}$ with $\mathsf{Name}(o'_1) = \text{Jon}$, $\mathsf{Name}(o'_2) = \text{Mary}$. Now string matching does not say too much: it may happen that $o_1$ and $o'_1$ are the same object (think of a typo in the dataset), while $o_2$ and $o'_2$ are different (although equally named) objects. Of course, for better matching we could use full names or ID numbers or something similar (called, in the database parlance, primary keys), but absolutely reliable keys are rare, and typos and bugs can compromise them anyway. Thus, for object structures that Bx needs to keep in sync, deltas between models need to be independently specified, e.g., by specifying a *sameness relation* $u \subset B \times B'$ between models. For example, $u = \{o_1, o'_1\}$ says that John@$B$ and Jon@$B'$ are the same person while Mary@$B$ and Mary@$B'$ are not. Hence, model spaces in Bx are categories (objects are models and arrows are update/delta specifications) rather than sets (codiscrete categories).

#### 2.2 Consistency restoration via update propagation: An Example

Figure 1 presents a simple example of delta propagation for consistency restoration. Models consist of objects (in the sense of OO programming) with attributes (a.k.a. labelled records), e.g., the source model $A$ consists of three objects identified by their oids (object identifiers) #A, #J, #M (think about employees of some company) with attribute values as shown in the table: attribute $\mathsf{Expr.}$ refers to Experience measured by a number of years, and $\mathsf{Depart.}$ is the column of department names. The schema of the table, i.e., the triple $S_{\mathbf{A}}$ of attributes ($\mathsf{Name}$, $\mathsf{Expr.}$, $\mathsf{Depart.}$) with their domains of values ***String***, ***Integer***, ***String*** resp., determines a model space $\mathbf{A}$. A model $X \in \mathbf{A}$ is given by its set of objects $\mathsf{OID}^X$ together with three functions $\mathsf{Name}^X$, $\mathsf{Expr.}^X$, $\mathsf{Depart.}^X$ from the same domain $\mathsf{OID}^X$ to targets ***String***, ***Integer***, ***String*** resp., which are compactly specified by tables as shown for model $A$. The target model space $\mathbf{B}$ is given by a similar schema $S_{\mathbf{B}}$ consisting of two attributes. The $\mathbf{B}$-view $\mathsf{get}(X)$ of an $\mathbf{A}$-model $X$ is computed by selecting those oids $\#O \in \mathsf{OID}^X$ for which $\mathsf{Depart.}^X(\#O)$ is an *IT-department*, i.e., an element of the set $IT \stackrel{\text{def}}{=} \{\text{ML}, \text{DB}\}$. For example, the upper part of the figure shows the IT-view $B$ of model $A$.

We assume that all column names in schemas $S_{\mathbf{A}}$, and $S_{\mathbf{B}}$ are qualified by schema names, e.g., OID@$S_{\mathbf{A}}$, OID@$S_{\mathbf{B}}$ etc, so that schemas are disjoint except elementary domains like ***String*** etc. Also disjoint are OID-values, e.g., #J@$A$ and #J@$B$ are different elements, but constants like John and Mary are elements of set ***String*** shared by both schemas. To shorten long expressions in the diagrams, we will often omit qualifiers and write $\#J = \#J$ meaning $\#J@A = \#J@B$ or $\#J@B = \#J@B'$ depending on the context given by the diagram; often we will also write $\#J$ and $\#J'$ for such OIDs. Also, when we write $\#J = \#J$ inside block arrows denoting updates, we actually mean a pair, e.g., $(\#J@B, \#J@B')$.

Given two models over the same schema, say, $B$ and $B'$ over $S_{\mathbf{B}}$, an update $v\colon B \to B'$ is a relation $v \subset \mathsf{OID}^{B} \times \mathsf{OID}^{B'}$; if a schema contains several nodes, an update should provide a relation $v_N$ for each node $N$ in the schema.

Note an essential difference between the two parallel updates $v_1, v_2\colon B \to B'$ specified in the figure. Update $v_1$ says that John's name was changed to Jon (think of fixing a typo), and the experience data for Mary were also corrected (either because of a typo or, e.g., because the department started to use a new ML method for which Mary has a longer experience). Update $v_2$ specifies the same story for John but a new story for Mary: it says that Mary $\#M$ left the IT-view and Mary $\#M'$ is a new employee in one of IT-departments.

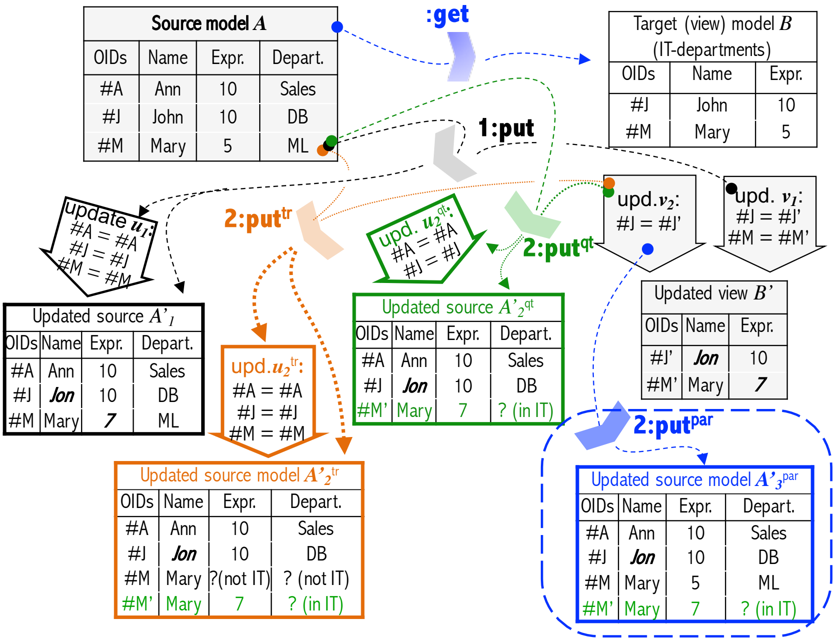

Fig. 1: Example of update propagation

### 2.3 Update propagation and update policies

The updated view $B'$ is inconsistent with the source $A$ and the latter is to be updated accordingly — we say that update $v$ is to be propagated back to $A$. Propagation of $v_1$ is easy: we just update accordingly the values of the attributes as shown in the figure in the block arrow $u_1\colon A \to A'_1$ (of black colour). Importantly, propagation needs two pieces of data: the view update $v_1$ and the original state $A$ of the source as shown in the figure by two data-flow lines into the chevron 1:$\mathsf{put}$; the latter denotes invocation of the backward propagation operation $\mathsf{put}$ (read "put view update back to the source"). The quadruple $1 = (v_1, A,\ u_1, A')$ can be seen as an *instance* of operation $\mathsf{put}$, hence the notation 1:$\mathsf{put}$ (borrowed from the UML).

Propagation of update $v_2$ is more challenging: Mary can disappear from the IT-view because a) she quit the company, b) she transitioned to a non-IT department, and c) the view definition has changed, e.g., the new view must only show employees with experience more than 5 years. Choosing between these possibilities is often called choosing an *(update) policy*. We will consider the case of changing the view in Sect. 3, and in the current section discuss policies a) and b) (ignore for a while the propagation scenario shown in blue in the right lower corner of the figure that shows policy c)).

For policy a), further referred to as *quiting* and briefly denoted by $\mathsf{qt}$, the result of update propagation is shown in the figure with green colour: notice the update (block) arrow $u_2^{\mathsf{qt}}$ and its result, model $A_2^{'\mathsf{qt}}$, produced by invoking operation $\mathsf{put}^{\mathsf{qt}}$. Note that while we know the new employee Mary works in one of IT departments, we do not know in which one. This is specified with a special value '?' (a.k.a. labelled null in the database parlance).

For policy b), further referred to as *transition* and denoted $\mathsf{tr}$, the result of update propagation is shown in the figure with orange colour: notice update arrow $u_2^{\mathsf{tr}}$ and its result, model $A_2^{'\mathsf{tr}}$ produced by $\mathsf{put}^{\mathsf{tr}}$. Mary #M is the old employee who transitioned to a new non-IT department, for which her expertize is unknown. Mary #M' is a new employee in one of IT-departments (we assume that the set of departments is not exhausted by those appearing in a particular state $A \in \mathbf{A}$). There are also updates whose backward propagation is uniquely defined and does not need a policy, e.g., update $v_1$ is such.

An important property of update propagations we have considered is that they restore consistency: the view of the updated source equals to the updated view initiated the update: $\mathsf{get}(A') = B'$; moreover, this equality extends for update arrows: $\mathsf{get}(u_i) = v_i$, $i = 1, 2$. Such extensions can be derived from view definitions if the latter are determined by so called monotonic queries (which encompass a wide class of practically useful queries including the Select-Project-Join class). For views defined by non-monotonic queries, in order to obtain $\mathsf{get}$'s action on source updates $u\colon A \to A'$, a suitable policy is to be added to the view definition (see [1,14,12] for details and discussion). Moreover, normally $\mathsf{get}$ preserves identity updates, $\mathsf{get}(\mathsf{id}_A) = \mathsf{id}_{\mathsf{get}(A)}$, and update composition: for any $u\colon A \to A'$ and $u'\colon A' \to A''$, equality $\mathsf{get}(u; u') = \mathsf{get}(u); \mathsf{get}(u')$ holds.

#### 2.4 Delta lenses

Our discussion of the example can be summarized in the following algebraic terms. We have two categories of *models* and *updates*, $\mathbf{A}$ and $\mathbf{B}$, and a functor $\mathsf{get}\colon \mathbf{A} \to \mathbf{B}$ incrementally computing $\mathbf{B}$-views of $\mathbf{A}$-models (we will often write $A.\mathsf{get}$ for $\mathsf{get}(A)$). We also suppose that for a chosen update policy, we have worked out precise procedures for how to propagate any view update backwards. This gives us a family of operations $\mathsf{put}_A : \mathbf{A}(A, \_) \leftarrow \mathbf{B}(A.\mathsf{get}, \_)$ indexed by $\mathbf{A}$-objects, $A \in \mathbf{A}_0$, for which we write $\mathsf{put}_A.v$ or $\mathsf{put}_A(v)$ interchangeably.

**Definition 1 (Delta Lenses ([12]))** Let $\mathbf{A}$, $\mathbf{B}$ be two categories. An *(asymmetric delta) lens* from $\mathbf{A}$ (the source of the lens) to $\mathbf{B}$ (the target) is a pair $\ell = (\mathsf{get}, \mathsf{put})$, where $\mathsf{get}\colon \mathbf{A} \to \mathbf{B}$ is a functor and $\mathsf{put}$ is a family of operations $\mathsf{put}_A : \mathbf{A}(A, \_) \leftarrow \mathbf{B}(A.\mathsf{get}, \_)$ indexed by objects of $\mathbf{A}$, $A \in \mathbf{A}_0$. Given $A$, operation $\mathsf{put}_A$ maps any arrow $v\colon A.\mathsf{get} \to B'$ to an arrow $u\colon A \to A'$ such that $A'.\mathsf{get} = B'$. The last condition is called (co)discrete Putget law:

$$(\mathsf{Putget})_0 \qquad (\mathsf{put}_A.v).\mathsf{cod}.\mathsf{get}_0 = v.\mathsf{cod} \text{ for all } A \in \mathbf{A}_0 \text{ and } v \in \mathbf{B}(A.\mathsf{get}, \_)$$

where $\mathsf{get}_0$ denotes the object function of functor $\mathsf{get}$. We will write a lens as an arrow $\ell\colon \mathbf{A} \to \mathbf{B}$ going in the direction of $\mathsf{get}$.

Note that family $\mathsf{put}$ corresponds to a chosen update policy, e.g., in terms of the example above, for the same view functor $\mathsf{get}$, we have two families of $\mathsf{put}$-operations, $\mathsf{put}^{\mathsf{qt}}$ and $\mathsf{put}^{\mathsf{tr}}$, corresponding to the two updated policies we discussed. These two policies determine two lenses $\ell^{\mathsf{qt}} = (\mathsf{get}, \mathsf{put}^{\mathsf{qt}})$ and $\ell^{\mathsf{tr}} = (\mathsf{get}, \mathsf{put}^{\mathsf{tr}})$ sharing the same $\mathsf{get}$.

**Definition 2 (Well-behavedness)** A *(lens) equational law* is an equation to hold for all values of two variables: $A \in \mathbf{A}_0$ and $v\colon A.\mathsf{get} \to T'$. A lens is called *well-behaved (wb)* if the following two laws hold:

$$(\mathsf{Stability}) \qquad \mathsf{id}_A = \mathsf{put}_A.\mathsf{id}_{A.\mathsf{get}} \text{ for all } A \in \mathbf{A}_0$$

$$(\mathsf{Putget}) \qquad (\mathsf{put}_A.v).\mathsf{get} = v \text{ for all } A \in \mathbf{A}_0 \text{ and all } v \in \mathbf{B}(A.\mathsf{get}, \_)$$

*Remark 1.* Stability law says that a wb lens does nothing if nothing happens on the target side (no actions without triggers). Putget requires consistency after the backward propagation is finished. Note the distinction between the $\mathrm{Putget}_0$ condition included into the very definition of a lens, and the full Putget law required for the wb lenses. The former is needed to ensure smooth tiling of $\mathsf{put}$-squares (i.e., arrow squares describing application of $\mathsf{put}$ to a view update and its result) both horizontally (for sequential composition) and vertically (not considered in the paper). The full Putget assures true consistency as considering a state $B'$ alone does not say much about the real update and elements of $B'$ cannot be properly interpreted. The real story is specified by delta $v\colon B \to B'$, and consistency restoration needs the full ($\mathsf{PutGet}$) law as above. [2]

A more detailed trailer of lenses can be found in the long version [9].

[2] As shown in [6], the $\mathrm{Putget}_0$ condition is needed if we want to define operations $\mathsf{put}$ separately from the functor $\mathsf{get}$: then we still need a function $\mathsf{get}_0\colon \mathbf{A}_0 \to \mathbf{B}_0$ and the codiscrete Putget law to ensure a reasonable behaviour of $\mathsf{put}$.

## 3 Asymmetric Learning Lenses with Amendments

We will begin with a brief motivating discussion, and then proceed with formal definitions

### 3.1 Does Bx need categorical learning?

Enriching delta lenses with learning capabilities has a clear practical sense for Bx. Having a lens $(\mathsf{get}, \mathsf{put})\colon \mathbf{A} \to \mathbf{B}$ and inconsistency $A.\mathsf{get} \neq B'$, the idea of learning extends the notion of the search space and allows us to update the transformation itself so that the final consistency is achieved for a new transformation $\mathsf{get}'\colon A.\mathsf{get}' = B'$. For example, in the case shown in Fig. 1, disappearance of Mary #M in the updated view $B'$ can be caused by changing the view definition, which now requires to show only those employees whose experience is more than 5 years and hence Mary #M is to be removed from the view, while Mary #M' is a new IT-employee whose experience satisfies the new definition. Then the update $v_2$ can be propagated as shown in the bottom right corner of Fig. 1, where index $\mathsf{par}$ indicates a new update policy allowing for view definition (parameter) change.

To manage the extended search possibilities, we parameterize the space of transformations as a family of mappings $\mathsf{get}_p\colon \mathbf{A} \to \mathbf{B}$ indexed over some parameter space $p \in \mathbf{P}$. For example, we may define the IT-view to be parameterized by the experience of employees shown in the view (including *any* experience as a special parameter value). Then we have two interrelated propagation operations that map an update $B \rightsquigarrow B'$ to a parameter update $p \rightsquigarrow p'$ and a source update $A \rightsquigarrow A'$. Thus, the extended search space allows for new update policies that look for updating the parameter as an update propagation possibility. The possibility to update the transformation appears to be very natural in at least two important Bx scenarios: a) model transformation design and b) model transformation evolution (cf. [21]), which necessitates the enrichment of the delta lens framework with parameterization and learning. Note that all transformations $\mathsf{get}_p$, $p \in \mathbf{P}$ are to be elements of the same lens, and operations $\mathsf{put}$ are *not* indexed by $p$, hence, formalization of learning by considering a family of ordinary lenses would *not* do the job.

**Categorical vs. codiscrete learning** Suppose that the parameter $p$ is itself a set, e.g., the set of departments forming a view can vary depending on some context. Then an update from $p$ to $p'$ has a relational structure as discussed above, i.e., $e\colon p \to p'$ is a relation $e \subset p{\times}p'$ specifying which departments disappeared from the view and which are freshly added. This is a general phenomenon: as soon as parameters are structures (sets of objects or graphs of objects and attributes), a parameter change becomes a structured delta and the space of parameters gives rise to a category $\mathbf{P}$. The search/propagation procedure returns an arrow $e\colon p \to p'$ in this category, which updates the parameter value from $p$ to $p'$. Hence, a general model of supervised learning should assume $\mathbf{P}$ to be a category (and we say that learning is *categorical*). The case of the parameter space being a set is captured by considering a codiscrete category $\mathbf{P}$ whose only arrows are pairs of its objects; we call such learning *codiscrete*.

#### 3.2 Ala-lenses

The notion of a *parameterized functor (p-functor)* is fundamental for ala-lenses, but is not a lens notion per se and is thus placed into Appendix Sect. A.1. We will work with its exponential (rather than equivalent product-based) formulation but will do uncurrying and currying back if necessary, and often using the same symbol for an arrow $f$ and its uncurried version $\check{f}$.

**Definition 3 (ala-lenses)** Let $\mathbf{A}$ and $\mathbf{B}$ be categories. An *ala-lens* from $\mathbf{A}$ (the *source* of the lens) to $\mathbf{B}$ (the *target*) is a pair $\ell = (\mathsf{get}, \mathsf{put})$ whose first component is a p-functor $\mathsf{get}\colon \mathbf{A} \xrightarrow{\mathbf{P}} \mathbf{B}$ and the second component is a triple of (families of) operations $\mathsf{put} = (\mathsf{put}^{\mathsf{upd}}_{p,A}, \mathsf{put}^{\mathsf{req}}_{p,A}, \mathsf{put}^{\mathsf{self}}_{p,A})$ indexed by pairs $p \in \mathbf{P}_0$, $A \in \mathbf{A}_0$; arities of the operations are specified below after we introduce some notation. Names $\mathsf{req}$ (for 'request') and $\mathsf{upd}$ (for 'update') are chosen to match the terminology in [17].

Categories $\mathbf{A}$, $\mathbf{B}$ are called *model spaces*, their objects are *models* and their arrows are *(model) updates* or *deltas*. Objects of $\mathbf{P}$ are called *parameters* and are denoted by small letters $p, p', ..$ rather than capital ones to avoid confusion with [17], in which capital $P$ is used for the entire parameter set. Arrows of $\mathbf{P}$ are called *parameter deltas*. For a parameter $p \in \mathbf{P}_0$, we write $\mathsf{get}_p$ for the functor $\mathsf{get}(p)\colon \mathbf{A} \to \mathbf{B}$ (read "get $\mathbf{B}$-views of $\mathbf{A}$"), and if $A \in \mathbf{A}_0$ is a source model, its $\mathsf{get}_p$-view is denoted by $\mathsf{get}_p(A)$ or $A.\mathsf{get}_p$ or even $A_p$ (so that $\_{}_p$ becomes yet another notation for functor $\mathsf{get}_p$). Given a parameter delta $e\colon p \to p'$ and a source model $A \in \mathbf{A}_0$, the model delta $\mathsf{get}(e)\colon \mathsf{get}_p(A) \to \mathsf{get}_{p'}(A)$ will be denoted by $\mathsf{get}_e(A)$ or $e_S$ (rather than $A_e$ as we would like to keep capital letters for objects only). In the uncurried version, $\mathsf{get}_e(A)$ is nothing but $\check{\mathsf{get}}(e, \mathsf{id}_S)$

Since $\mathsf{get}_e$ is a natural transformation, for any delta $u\colon A \to A'$ we have a commutative square $e_S; u_{p'} = u_p; e_{A'}$ (whose diagonal is $\check{\mathsf{get}}(e, u)$). We will denote the diagonal of this square by $u.\mathsf{get}_e$ or $u_e\colon A_p \to A'_{p'}$. Thus, we use notation

$$
(1)\qquad
\begin{aligned}
&A_p \overset{\text{def}}{=} A.\mathsf{get}_p \overset{\text{def}}{=} \mathsf{get}_p(A) \overset{\text{def}}{=} \mathsf{get}(p)(A)\\
&u_e \overset{\text{def}}{=} u.\mathsf{get}_e \overset{\text{def}}{=} \mathsf{get}_e(u) \overset{\text{def}}{=} \mathsf{get}(e)(u) \overset{\text{def}}{=} e_S; u_{p'} \overset{\text{nat}}{=} u_p; e_{A'}\colon A_p \to A'_{p'}
\end{aligned}
$$

Now we describe operations $\mathsf{put}$. They all have the same indexing set $\mathbf{P}_0 \times \mathbf{A}_0$, and the same domain: for any index $p, A$ and any model delta $v\colon A_p \to B'$ in $\mathbf{B}$, the value $\mathsf{put}^{\mathbf{x}}_{p,A}(p, A)$, $\mathbf{x} \in \{\mathsf{req}, \mathsf{upd}, \mathsf{self}\}$ is defined and unique:

$$
(2)\qquad
\begin{array}{ll}
\mathsf{put}^{\mathsf{upd}}_{p,A}\colon p \to p' & \text{is a parameter delta from } p,\\
\mathsf{put}^{\mathsf{req}}_{p,A}\colon A \to A' & \text{is a model delta from } A,\\
\mathsf{put}^{\mathsf{self}}_{p,A}\colon B' \to A'_{p'} & \text{is a model delta from } B'\\
& \text{called the } \textit{amendment} \text{ and denoted by } v^{@}.
\end{array}
$$

Note that the definition of $\mathsf{put}^{\mathsf{self}}$ involves an equational dependency between all three operations: for all $A \in \mathbf{A}_0$, $v \in \mathbf{B}(A.\mathsf{get}, \_)$, we require

$$(\mathsf{Putget})_0 \qquad (\mathsf{put}^{\mathsf{req}}_A.v).\mathsf{cod}.\mathsf{get}_{p'} = (v; \mathsf{put}^{\mathsf{self}}_A).\mathsf{cod} \text{ where } p' = (\mathsf{put}^{\mathsf{upd}}_A.v).\mathsf{cod}$$

We will write an ala-lens as an arrow $\ell = (\mathsf{get}, \mathsf{put})\colon \mathbf{A} \xrightarrow{\mathbf{P}} \mathbf{B}$.

A lens is called *(twice) codiscrete* if categories $\mathbf{A}$, $\mathbf{B}$, $\mathbf{P}$ are codiscrete and thus $\mathsf{get}\colon \mathbf{A} \xrightarrow{\mathbf{P}} \mathbf{B}$ is a parameterized function. If only $\mathbf{P}$ is codiscrete, we call $\ell$ a *codiscretely learning* delta lens, while if only model spaces are codiscrete, we call $\ell$ a *categorically learning* codiscrete lens.

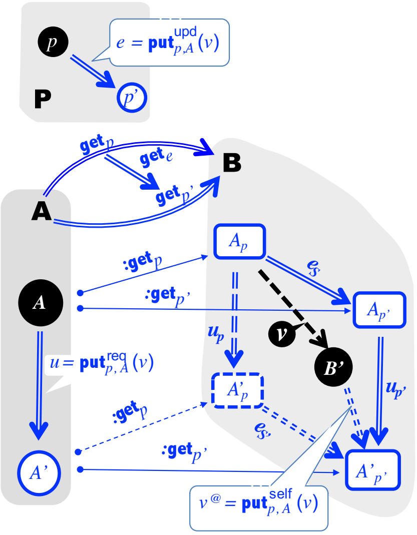

Fig. 2: Ala-lens operations

Diagram in Fig. 2 shows how a lens' operations are interrelated. The upper part shows an arrow $e\colon p \to p'$ in category $\mathbf{P}$ and two corresponding functors from $\mathbf{A}$ to $\mathbf{B}$. The lower part is to be seen as a 3D-prism with visible front face $AA_{p'}A'_{p'}A'$ and visible upper face $AA_pA_{p'}$, the bottom and two back faces are invisible, and the corresponding arrows are dashed. The prism denotes an algebraic term: given elements are shown with black fill and white font while derived elements are blue (recalls being mechanically computed) and blank (double-body arrows are considered as "blank"). The two pairs of arrows originating from $A$ and $A'$ are not blank because they denote pairs of nodes (the UML says *links*) rather than mappings/deltas between nodes. Equational definitions of deltas $e, u, v^@$ are written up in the three callouts near them. The right back face of the prism is formed by the two vertical derived deltas $u_p = u.\mathsf{get}_p$ and $u_{p'} = u.\mathsf{get}_{p'}$, and the two matching them horizontal derived deltas $e_S = \mathsf{get}_e(A)$ and $e_{A'} = \mathsf{get}_e(A')$; together they form a commutative square due to the naturality of $\mathsf{get}(e)$ as explained earlier.

**Definition 4 (Well-behavedness)** An ala-lens is called *well-behaved (wb)* if the following two laws hold for all $p \in \mathbf{P}_0$, $A \in \mathbf{A}_0$ and $v\colon A_p \to B'$:

(Stability) if $v = \mathsf{id}_{A_p}$ then all three propagated updates $e, u, v^@$ are identities:

$$\mathsf{put}^{\mathsf{upd}}_{p,A}(\mathsf{id}_{A_p}) = \mathsf{id}_p, \quad \mathsf{put}^{\mathsf{req}}_{p,A}(\mathsf{id}_{A_p}) = \mathsf{id}_S, \quad \mathsf{put}^{\mathsf{self}}_{p,A}(\mathsf{id}_{A_p}) = \mathsf{id}_{A_p}$$

$$(\mathsf{Putget}) \qquad (\mathsf{put}^{\mathsf{req}}_{p,A}.v).\mathsf{get}_e = v; v^@ \text{ where } e = \mathsf{put}^{\mathsf{upd}}_{p,A}(v) \text{ and } v^@ = \mathsf{put}^{\mathsf{self}}_{p,A}(v)$$

*Remark 2.* Note that Remark 1 about the Putget law is again applicable.

*Example 1 (Identity lenses).* Any category $\mathbf{A}$ gives rise to an ala-lens $\mathcal{id}_{\mathbf{A}}$ with the following components. The source and target spaces are equal to $\mathbf{A}$, and the parameter space is $\mathbf{1}$. Functor $\mathsf{get}$ is the identity functor and all $\mathsf{put}$s are identities. Obviously, this lens is wb.

*Example 2 (Iso-lenses).* Let $\iota$: $\mathbf{A} \to \mathbf{B}$ be an isomorphism between model spaces. It gives rise to a wb ala-lens $\ell(\iota)$: $\mathbf{A} \to \mathbf{B}$ with $\mathbf{P}^{\ell(\iota)} = \mathbf{1} = \{*\}$ as follows. Given any $A$ in $\mathbf{A}$ and $v$: $\iota(A) \to B'$ in $\mathbf{B}$, we define $\mathsf{put}^{\ell(\iota).\mathsf{req}}_{*,A}(v) = \iota^{-1}(v)$ while the two other put operations map $v$ to identities.

*Example 3 (Bx lenses).* Examples of wb aa-lenses modelling a Bx can be found in [11]: they all can be considered as ala-lenses with a trivial parameter space $\mathbf{1}$.

*Example 4 (Learners).* Learners defined in [17] are codiscretely learning codiscrete lenses with amendment, and as such satisfy (the amended) Putget (Remark 1). Looking at the opposite direction, ala-lenses are a categorification of learners as detailed in Fig. 8 on p. 194.

## 4 Compositionality of ala-lenses

This section explores the compositional structure of the universe of ala-lenses; especially interesting is their sequential composition. We will begin with a small example demonstrating sequential composition of ordinary lenses and showing that the notion of update policy transcends individual lenses. Then we define sequential and parallel composition of ala-lenses (the former is much more involved than for ordinary lenses) and show that wb ala-lenses can be organized into an sm-category. Finally, we formalize the notion of a compositional update policy via the notion of a compositional bidirectional language.

### 4.1 Compositionality of update policies: An example

Fig. 3 extends the example in Fig. 1 with a new model space $\mathbf{C}$ whose schema consists of the only attribute $\mathsf{Name}$, and a view of the IT-view, in which only employees of the ML department are to be shown. Thus, we now have two functors, $\mathsf{get1}$: $\mathbf{A} \to \mathbf{B}$ and $\mathsf{get2}$: $\mathbf{B} \to \mathbf{C}$, and their composition $\mathsf{Get}$: $\mathbf{A} \to \mathbf{C}$ (referred to as the *long* get). The top part of Fig. 3 shows how it works for model $A$ considered above.

Each of the two policies, policy $\mathsf{qt}$ (green) and policy $\mathsf{tr}$ (orange), in which person's disappearance from the view are interpreted, resp., as quiting the company and transitioning to a department not included into the view, is applicable to the new view mappings $\mathsf{get2}$ and $\mathsf{Get}$, thus giving us six lenses shown in Fig. 4 with solid arrows; amongst them, lenses, $\mathcal{L}^{\mathsf{qt}}$ and $\mathcal{L}^{\mathsf{tr}}$ are obtained by applying policy *pol* to the (long) functor $\mathsf{Get}$;, and we will refer to them *long* lenses. In addition, we can compose lenses of the same colour as shown in Fig. 4 by dashed arrows (and we can also compose lenses of different colours ($\ell_1^{\mathsf{qt}}$ with $\ell_2^{\mathsf{tr}}$ and $\ell_1^{\mathsf{tr}}$ with $\ell_2^{\mathsf{qt}}$) but we do not need them). Now an important question is how long and composed lenses are related: whether $\mathcal{L}^{pol}$ and $\ell_1^{pol};\ell_2^{pol}$ for $pol \in \{\mathsf{qt}, \mathsf{tr}\}$, are equal (perhaps up to some equivalence) or different?

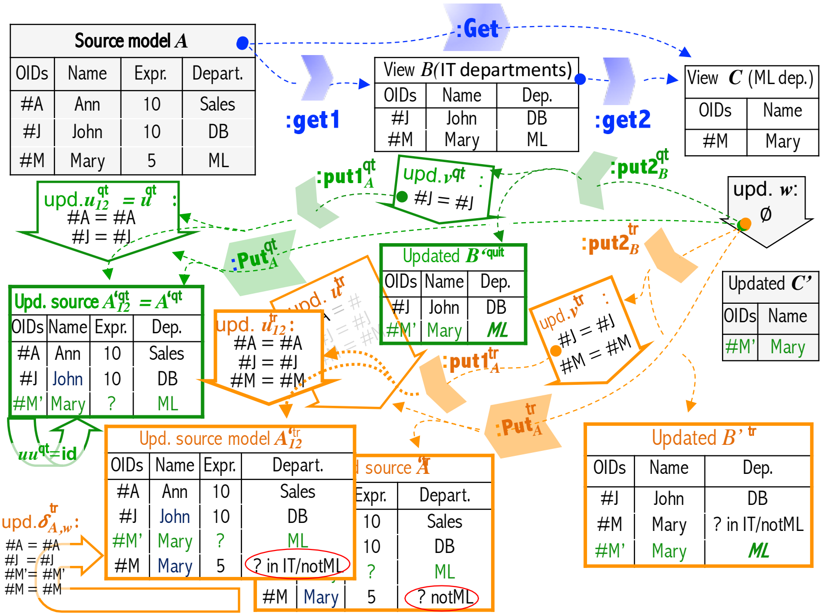

Fig. 3: Example cont'd: functoriality of update policies

Fig. 3 demonstrates how the mechanisms work with a simple example. We begin with an update $w$ of the view $C$ that says that Mary $\#M$ left the ML department, and a new Mary $\#M'$ was hired for ML. Policy $\mathsf{qt}$ interprets Mary's disappearance as quiting the company, and hence this Mary doesn't appear in view $B'^{\mathsf{qt}}$ produced by $\mathsf{put2}^{\mathsf{qt}}$ nor in view $A'^{\mathsf{qt}}_{12}$ produced from $B'^{\mathsf{qt}}$ by $\mathsf{put1}^{\mathsf{qt}}$, and updates $v^{\mathsf{qt}}$ and $u^{\mathsf{qt}}_{12}$ are written accordingly. Obviously, Mary also does not appear in view $A'^{\mathsf{qt}}$ produced by the long lens's $\mathsf{Put}^{\mathsf{qt}}$. Thus, $\mathsf{put1}^{\mathsf{qt}}_A(\mathsf{put2}^{\mathsf{qt}}_A(w)) = \mathsf{Put}^{\mathsf{qt}}_A(w)$, and it is easy to understand that such equality will hold for any source model $A$ and any update $w\colon C \to C'$ due to the nature of our two views $\mathsf{get1}$ and $\mathsf{get2}$. Hence, $\mathcal{L}^{\mathsf{qt}} = \ell^{\mathsf{qt}}_1; \ell^{\mathsf{qt}}_2$ where $\mathcal{L}^{\mathsf{qt}} = (\mathsf{Get}, \mathsf{Put}^{\mathsf{qt}})$ and $\ell^{\mathsf{qt}}_i = (\mathsf{get}i, \mathsf{put}i^{\mathsf{qt}})$.

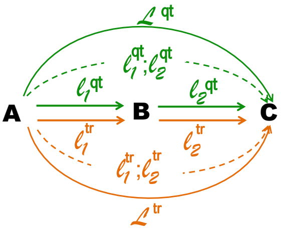

Fig. 4: Lens combination schemas for Fig. 3

The situation with policy $\mathsf{tr}$ is more interesting. Model $A'^{\mathsf{tr}}_{12}$ produced by the composed lens $\ell^{\mathsf{tr}}_1; \ell^{\mathsf{tr}}_2$, and model $A'^{\mathsf{tr}}$ produced by the long lens $\mathcal{L}^{\mathsf{tr}} = (\mathsf{Get}, \mathsf{Put}^{\mathsf{tr}})$ are different as shown in the figure (notice the two different values for Mary's department framed with red ovals in the models). Indeed, the composed lens has more information about the old employee Mary—it knows that Mary was in the IT view, and hence can propagate the update more accurately. The comparison update $\delta^{\mathsf{tr}}_{A,w}\colon A'^{\mathsf{tr}} \to A'^{\mathsf{tr}}_{12}$ adds this missing information so that equality $u^{\mathsf{tr}}; \delta^{\mathsf{tr}}_{A,w} = u^{\mathsf{tr}}_{12}$ holds. This is a general phenomenon: functor composition looses information and, in general, functor $\mathsf{Get} = \mathsf{get1}; \mathsf{get2}$ knows less than the pair $(\mathsf{get1}, \mathsf{get2})$. Hence, operation $\mathsf{Put}$ back-propagating updates over $\mathsf{Get}$ (we will also say *inverting* Get) will, in general, result in less certain models than composition $\mathsf{put1} \circ \mathsf{put2}$ that inverts the composition $\mathsf{get1};\mathsf{get2}$ (a discussion and examples of this phenomenon in the context of vertical composition of updates can be found in [8]). Hence, comparison updates such as $\delta^{\mathsf{tr}}_{A,w}$ should exist for any $A$ and any $w\colon A.\mathsf{Get} \to C'$, and together they should give rise to something like a natural transformation between lenses, $\delta^{\mathsf{tr}}_{\mathbf{A},\mathbf{B},\mathbf{C}}\colon \mathscr{L}^{\mathsf{tr}} \Rightarrow \ell^{\mathsf{tr}}_1;\ell^{\mathsf{tr}}_2$. To make this notion precise, we need a notion of natural transformation between "functors" $\mathsf{put}$, which we leave for future work. In the present paper, we will consider policies like $\mathsf{qt}$, for which strict equality holds.

#### 4.2 Sequential composition of ala-lenses

Let $\mathscr{k}\colon \mathbf{A} \to \mathbf{B}$ and $\ell\colon \mathbf{B} \to \mathbf{C}$ be two ala-lenses with parameterized functors $\mathsf{get}^{\mathscr{k}}\colon \mathbf{P} \to [\mathbf{A},\mathbf{B}]$ and $\mathsf{get}^{\ell}\colon \mathbf{Q} \to [\mathbf{B},\mathbf{C}]$ resp. Their *composition* is the following ala-lens $\mathscr{k};\ell$. Its parameter space is the product $\mathbf{P} \times \mathbf{Q}$, and the $\mathsf{get}$-family is defined as follows. For any pair of parameters $(p,q)$ (we will write $pq$), $\mathsf{get}^{\mathscr{k};\ell}_{pq} = \mathsf{get}^{\mathscr{k}}_p;\mathsf{get}^{\ell}_q\colon \mathbf{A} \to \mathbf{C}$. Given a pair of parameter deltas, $e\colon p \to p'$ in $\mathbf{P}$ and $h\colon q \to q'$ in $\mathbf{Q}$, their $\mathsf{get}^{\mathscr{k};\ell}$-image is the Godement product $*$ of natural transformations, $\mathsf{get}^{\mathscr{k};\ell}(eh) = \mathsf{get}^{\mathscr{k}}(e) * \mathsf{get}^{\ell}(h)$ ( we will also write $\mathsf{get}^{\mathscr{k}}_e \,||\, \mathsf{get}^{\ell}_h$)

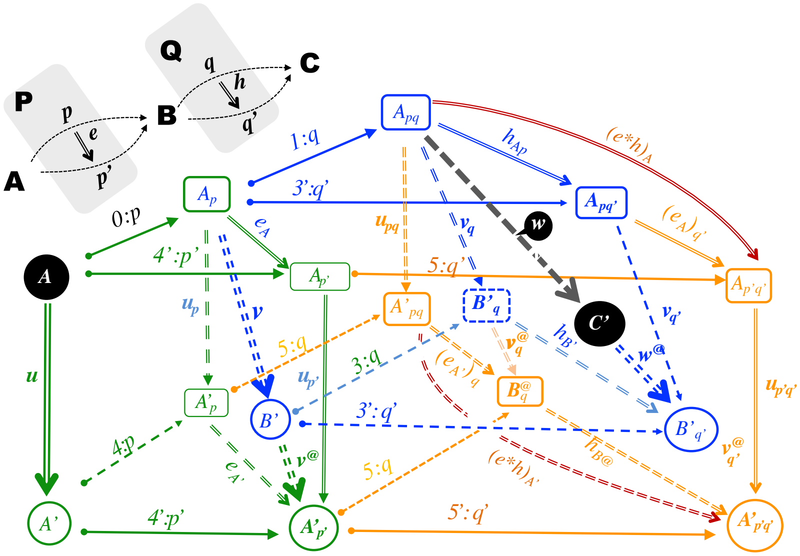

Fig. 5: Sequential composition of apa-lenses

Now we define $\mathscr{k};\ell$'s propagation operations $\mathsf{puts}$. Let $(A, pq, A_{pq})$ with $A \in \mathbf{A}_0$, $pq \in (\mathbf{P}\times\mathbf{Q})_0$, $A.\mathsf{get}^{\mathscr{k}}_p.\mathsf{get}^{\ell}_q = A_{pq} \in \mathbf{C}_0$ be a state of lens $\mathscr{k};\ell$, and $w\colon A_{pq} \to C'$ is a target update as shown in Fig. 3. For the first propagation step, we run lens $\ell$ as shown in Fig. 3 with the blue colour for derived elements: this is just an instantiation of the pattern of Fig. 2 with the source object being $A_p = A.\mathsf{get}_p$ and parameter $q$. The results are deltas

(3)
$$h = \mathsf{put}^{\ell.\mathsf{upd}}_{q,A_p}(w)\colon q \to q', v = \mathsf{put}^{\ell.\mathsf{req}}_{q,A_p}(w)\colon A_p \to B', w^{@} = \mathsf{put}^{\ell.\mathsf{self}}_{q,A_p}(w)\colon C' \to B'_{q'}.$$

Next we run lens $\text{ꝁ}$ at state $(p, A)$ and the target update $v$ produced by lens $\ell$; it is yet another instantiation of pattern in Fig. 2 (this time with the green colour for derived elements), which produces three deltas

(4)
$$e = \mathsf{put}^{\text{ꝁ}.\mathsf{upd}}_{p,A}(v)\colon p \to p', u = \mathsf{put}^{\text{ꝁ}.\mathsf{req}}_{p,A}(v)\colon A \to A', v^{@} = \mathsf{put}^{\text{ꝁ}.\mathsf{self}}_{p,A}(v)\colon B' \to A'_{p'}.$$

These data specify the green prism adjoint to the blue prism: the edge $v$ of the latter is the "first half" of the right back face diagonal $A_pA'_{p'}$ of the former. In order to make an instance of the pattern in Fig. 2 for lens $\text{ꝁ};\ell$, we need to extend the blue-green diagram to a triangle prism by filling-in the corresponding "empty space". These filling-in arrows are provided by functors $\mathsf{get}^{\ell}$ and $\mathsf{get}^{\text{ꝁ}}$ and shown in orange (where we have chosen one of the two equivalent ways of forming the Godement product – note two curve brown arrows). In this way we obtain yet another instantiation of the pattern in Fig. 2 denoted by $\text{ꝁ};\ell$:

(5) $$\mathsf{put}^{(\text{ꝁ};\ell)\mathsf{upd}}_{A,pq}(w) = (e, h), \quad \mathsf{put}^{(\text{ꝁ};\ell)\mathsf{req}}_{A,pq}(w) = u, \quad \mathsf{put}^{(\text{ꝁ};\ell)\mathsf{self}}_{A,pq}(w) = w^{@}; v^{@}_{q'}$$

where $v^{@}_{q'}$ denotes $v^{@}.\mathsf{get}_{q'}$. Thus, we built an ala-lens $\text{ꝁ};\ell$, which satisfies equation $\mathsf{Putget}_0$ by construction.

**Theorem 1 (Sequential composition and lens laws).** *Given ala-lenses $\text{ꝁ}\colon \mathbf{A} \to \mathbf{B}$ and $\ell\colon \mathbf{B} \to \mathbf{C}$, let lens $\text{ꝁ};\ell\colon \mathbf{A} \to \mathbf{C}$ be their sequential composition as defined above. Then the lens $\text{ꝁ};\ell$ is wb as soon as lenses $\text{ꝁ}$ and $\ell$ are such.*

See [9, Appendix A.3] for a proof.

#### 4.3 Parallel composition of ala-lenses

Let $\ell_i\colon \mathbf{A}_i \to \mathbf{B}_i$, $i = 1, 2$ be two ala-lenses with parameter spaces $\mathbf{P}_i$. The lens $\ell_1\|\ell_2\colon \mathbf{A}_1{\times}\mathbf{A}_2 \to \mathbf{B}_1{\times}\mathbf{B}_2$ is defined as follows. Parameter space $\ell_1\|\ell_2.\mathbf{P} = \mathbf{P}_1 \times \mathbf{P}_2$. For any pair $p_1\|p_2 \in (\mathbf{P}_1{\times}\mathbf{P}_2)_0$, define $\mathsf{get}^{\ell_1\|\ell_2}_{p_1\|p_2} = \mathsf{get}^{\ell_1}_{p_1} \times \mathsf{get}^{\ell_2}_{p_2}$ (we denote pairs of parameters by $p_1\|p_2$ rather than $p_1 \otimes p_2$ to shorten long formulas going beyond the page width). Further, for any pair of models $\in (\mathbf{A}_1 \times \mathbf{A}_2)_0$ and deltas $v_1\|v_2\colon ().\mathsf{get}^{\ell_1\|\ell_2}_{p_1\|p_2} \to B'_1\|B'_2$, we define componentwise

$$e = \mathsf{put}^{(\ell_1\|\ell_2)\mathsf{upd}}_{p_1\|p_2,}(v_1\|v_2)\colon p_1\|p_2 \to p'_1\|p'_2$$

by setting $e = e_1\|e_2$ where $e_i = \mathsf{put}^{\ell_i}_{p_i,S_i}(v_i)$, $i = 1, 2$ and similarly for $\mathsf{put}^{(\ell_1\|\ell_2)\mathsf{req}}_{p_1\|p_2,}$ and $\mathsf{put}^{(\ell_1\|\ell_2)\mathsf{self}}_{p_1\|p_2,}$ The following result is obvious.

**Theorem 2 (Parallel composition and lens laws).** *Lens $\ell_1\|\ell_2$ is wb as soon as lenses $\ell_1$ and $\ell_2$ are such.*

#### 4.4 Symmetric monoidal structure over ala-lenses

Our goal is to organize ala-lenses into an sm-category. To make sequential composition of ala-lenses associative, we need to consider them up to some equivalence (indeed, Cartesian product is not strictly associative).

**Definition 5 (Ala-lens Equivalence)** Two parallel ala-lenses $\ell, \hat{\ell}\colon \mathbf{A} \to \mathbf{B}$ are called *equivalent* if their parameter spaces are isomorphic via a functor $\iota\colon \mathbf{P} \to \hat{\mathbf{P}}$ such that for any $A \in \mathbf{A}_0$, $e\colon p \to p' \in \mathbf{P}$ and $v\colon (A.\mathsf{get}_p) \to T'$ the following holds (for $\mathbf{x} \in \{\mathsf{req}, \mathsf{self}\}$):

$$A.\mathsf{get}_e = A.\widehat{\mathsf{get}}_{\iota(e)},\ \iota(\mathsf{put}^{\mathsf{upd}}_{p,A}(v)) = \widehat{\mathsf{put}}_{\iota(p),A}(v),\ \text{ and } \mathsf{put}^{\mathbf{x}}_{p,A}(v) = \widehat{\mathsf{put}}^{\mathbf{x}}_{\iota(p),A}(v)$$

*Remark 3.* It would be more categorical to require delta isomorphisms (i.e., commutative squares whose horizontal edges are isomorphisms) rather than equalities as above. However, model spaces appearing in Bx-practice are skeletal categories (and even stronger than skeletal in the sense that all isos, including iso loops, are identities), for which isos become equalities so that the generality would degenerate into equality anyway.

It is easy to see that operations of lens' sequential and parallel composition are compatible with lens' equivalence and hence are well-defined for equivalence classes. Below we identify lenses with their equivalence classes by default.

**Theorem 3 (Ala-lenses form an sm-category).** *Operations of sequential and parallel composition of ala-lenses defined above give rise to an sm-category* ***aLaLens****, whose objects are model spaces (= categories) and arrows are (equivalence classes of) ala-lenses.* See [9, p.17 and Appendix A.4] for a proof.

#### 4.5 Functoriality of learning in the *delta* lens setting

As example in Sect. 4.1 shows, the notion of update policy transcends individual lenses. Hence, its proper formalization needs considering the entire category of ala-lenses and functoriality of a suitable mapping.

**Definition 6 (Bx-transformation language)** A *compositional bidirectional model transformation language* $\mathcal{L}_{\mathsf{bx}}$ is given by **(i)** an sm-category $\boldsymbol{pGet}(\mathcal{L}_{\mathsf{bx}})$ whose objects are *($\mathcal{L}_{\mathsf{bx}}$-)model spaces* and arrows are *($\mathcal{L}_{\mathsf{bx}}$-)transformations* which is supplied with forgetful functor into $\boldsymbol{pCat}$, and **(ii)** an sm-functor $L_{\mathcal{L}_{\mathsf{bx}}}\colon \boldsymbol{pGet}(\mathcal{L}_{\mathsf{bx}}) \to \boldsymbol{aLaLens}$ such that the lower triangle in the inset diagram commutes. (Forgetful functors in this diagram are named "$-X$" with $X$ referring to the structure to be forgotten.)

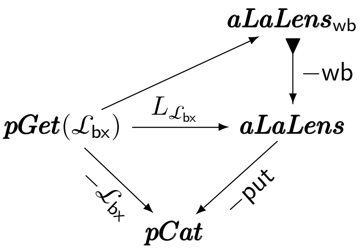

An $\mathcal{L}_{\mathsf{bx}}$-language is *well-behaved (wb)* if functor $L_{\mathcal{L}_{\mathsf{bx}}}$ factorizes as shown by the upper triangle of the diagram.

*Example.* A major compositionality result of Fong *et al* [17] states the existence of an sm-functor from the category of Euclidean spaces and parameterized differentiable functions (pd-functions) ***Para*** into the category ***Learn*** of learning algorithms (*learners*) as shown by the inset commutative diagram. (The functor is itself parameterized by a *step size* $0 < \varepsilon \in \mathbb{R}$ and an *error function* $\mathsf{err}\colon \mathbb{R}{\times}\mathbb{R} \to \mathbb{R}$ needed to specify the gradient descent procedure.) However, learners are nothing but codiscrete ala-lenses (see Sect. A.2), and thus the inset diagram is a codiscrete specialization of the diagram in Def. 6 above. That is, the category of Euclidean spaces and pd-functions, and the gradient descent method for back propagation, give rise to a (codiscrete) compositional bx-transformation language (over ***pSet*** rather than ***pCat***).

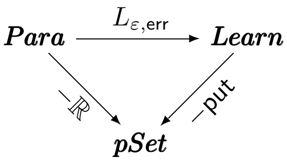

Finding a specifically Bx instance of Def. 6 (e.g., checking whether it holds for concrete languages and tools such as eMoflon [23] or groundTram [22]) is laborious and left for future work.

### 5 Related work

Figure 6 on the right is a simplified version of Fig. 8 on p. 194 convenient for our discussion here: immediate related work should be found in areas located at points (0,1) (codiscrete learning lenses) and (1,0) (delta lenses) of the plane. For the point (0,1), the paper [17] by Fong, Spivak and Tuyéras is fundamental: they defined the notion of a codiscrete learning lens (called a learner), proved a fundamental results about sm-functoriality of the gradient descent approach to ML, and thus laid a foundation for the compositional approach to change propagation with learning. One follow-up of that work is paper [16] by Fong and Johnson, in which they build an sm-functor ***Learn*** $\to$ ***sLens*** which maps learners to so called symmetric lenses. That paper is probably the first one where the terms 'lens' and 'learner' are met, but the initial observation that a learner whose parameter set is a singleton is actually a lens is due to Jules Hedges, see [16].

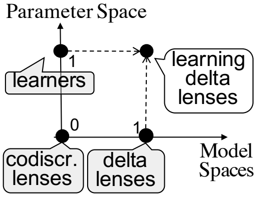

Fig. 6

There are conceptual and technical distinctions between [16] and the present paper. On the conceptual level, by encoding learners as symmetric lenses, they “hide” learning inside the lens framework and make it a technical rather than conceptual idea. In contrast, we consider parameterization and supervised learning as a fundamental idea and a first-class citizen for the lens framework, which grants creation of a new species of lenses. Moreover, while an ordinary lens is a way to invert a functor, a learning lens is a way to invert a parameterized functor so that learning lenses appear as an extension of the parameterization idea from functors to lenses. (This approach can probably be specified formally by treating parameterization as a suitably defined functorial construction.) Besides technical advantages (working with asymmetric lenses is simpler), our asymmetric model seems more adequate to the problem of learning functions rather than relations. On the technical level, the lens framework we develop in the paper is much more general than in [16]: we categorificated both the parameter space and model spaces, and we work with lenses with amendment (which allows us to relax the Putget law if needed).

As for the delta lens roots (the point (1,0) in the figure), delta lenses were motivated and formally defined in [12] (the asymmetric case) and [13] (the symmetric one). Categorical foundations for the delta lens theory were developed by Johnson and Rosebrugh in a series of papers (see [20] for references); this line is continued in Clarke's work [6]. The notion of a delta lens with amendments (in both asymmetric and symmetric variants) was defined in [11], and several composition results were proved. Another extensive body of work within the delta-based area is modelling and implementing model transformations with triple-graph grammars (TGG) [4,23]. TGG provide an implementation framework for delta lenses as is shown and discussed in [5,19,2], and thus inevitably consider change propagation on a much more concrete level than lenses. The author is not aware of any work considering functoriality of update policies developed within the TGG framework.

The present paper is probably the first one at the intersection (1,1) of the plane. The preliminary results have recently been reported at ACT'19 in Oxford to a representative lens community, and no references besides [17], [16] mentioned above were provided.

### 6 Conclusion

The perspective on Bx presented in the paper is an example of a fruitful interaction between two domains—ML and Bx. In order to be ported to Bx, the compositional approach to ML developed in [17] is to be categorificated as shown in Fig. 8 on p. 194. This opens a whole new program for Bx: checking that currently existing Bx languages and tools are compositional (and well-behaved) in the sense of Def. 6 p. 190. The wb compositionality is an important practical requirement as it allows for modular design and testing of bidirectional transformations. Surprisingly, but this important requirement has been missing from the agenda of the Bx community, e.g., the recent endeavour of developing an effective benchmark for Bx-tools [3] does not discuss it.

In a wider context, the main message of the paper is that the learning idea transcends its applications in ML: it is applicable and usable in many domains in which lenses are applicable such as model transformations, data migration, and open games [18]. Moreover, the categorificated learning may perhaps find useful applications in ML itself. In the current ML setting, the object to be learnt is a function $f\colon \mathbb{R}^m \to \mathbb{R}^n$ that, in the OO class modelling perspective, is a very simple structure: it can be seen as one object with a (huge) amount of attributes, or, perhaps, a predefined set of objects, which is not allowed to be changed during the search — only attribute values may be changed. In the delta lens view, such changes constitute a rather narrow class of updates and thus unjustifiably narrow the search space. Learning with the possibility to change dimensions $m, n$ may be an appropriate option in several contexts. On the other hand, while categorification of model spaces extends the search space, categorification of the parameter space would narrow the search space as we are allowed to replace a parameter $p$ by parameter $p'$ only if there is a suitable arrow $e\colon p \to p'$ in category $\mathbf{P}$. This narrowing may, perhaps, improve performance. All in all, the interaction between ML and Bx could be bidirectional!

## A Appendices

### A.1 Category of parameterized functors *pCat*

Category ***pCat*** has all small categories as objects. ***pCat***-arrows $\mathbf{A} \to \mathbf{B}$ are *parameterized* functors (*p-functors*) i.e., functors $f\colon \mathbf{P} \to [\mathbf{A}, \mathbf{B}]$ with $\mathbf{P}$ a small category of *parameters* and $[\mathbf{A}, \mathbf{B}]$ the category of functors from $\mathbf{A}$ to $\mathbf{B}$ and their natural transformations. For an object $p$ and an arrow $e\colon p \to p'$ in $\mathbf{P}$, we write $f_p$ for the functor $f(p)\colon \mathbf{A} \to \mathbf{B}$ and $f_e$ for the natural transformation $f(e)\colon f_p \Rightarrow f_{p'}$. We will write p-functors as labelled arrows $f\colon \mathbf{A} \xrightarrow{\mathbf{P}} \mathbf{B}$. As ***Cat*** is Cartesian closed, we have a natural isomorphism between ***Cat***$(\mathbf{P}, [\mathbf{A}, \mathbf{B}])$ and ***Cat***$(\mathbf{P}\times\mathbf{A}, \mathbf{B})$ and can reformulate the above definition in an equivalent way with functors $\mathbf{P}\times\mathbf{A} \to \mathbf{B}$. We prefer the former formulation as it corresponds to the notation $f\colon \mathbf{A} \xrightarrow{\mathbf{P}} \mathbf{B}$ visualizing $\mathbf{P}$ as a hidden state of the transformation, which seems adequate to the intuition of parameterized in our context. (If some technicalities may perhaps be easier to see with the product formulation, we will switch to the product view thus doing currying and uncurrying without special mentioning.) Sequential composition of of $f\colon \mathbf{A} \xrightarrow{\mathbf{P}} \mathbf{B}$ and $g\colon \mathbf{B} \xrightarrow{\mathbf{Q}} \mathbf{C}$ is $f.g\colon \mathbf{A} \xrightarrow{\mathbf{P}\times\mathbf{Q}} \mathbf{C}$ given by $(f.g)_{pq} \stackrel{\text{def}}{=} f_p.g_q$ for objects, i.e., pairs $p{\in}\mathbf{P}$, $q{\in}\mathbf{Q}$, and by the Godement product of natural transformations for arrows in $\mathbf{P}\times\mathbf{Q}$. That is, given a pair $e\colon p \to p'$ in $\mathbf{P}$ and $h\colon q \to q'$ in $\mathbf{Q}$, we define the transformation $(f.g)_{eh}\colon f_p.g_q \Rightarrow f_{p'}.g_{q'}$ to be the Godement product $f_e * g_h$.

Any category $\mathbf{A}$ gives rise to a p-functor $\mathsf{Id}_{\mathbf{A}}\colon \mathbf{A} \xrightarrow{\mathbf{1}} \mathbf{A}$, whose parameter space is a singleton category $\mathbf{1}$ with the only object $*$, $\mathsf{Id}_{\mathbf{A}}(*) = \mathsf{id}_{\mathbf{A}}$ and $\mathsf{Id}_A(\mathsf{id}_*)\colon \mathsf{id}_{\mathbf{A}} \Rightarrow \mathsf{id}_{\mathbf{A}}$ is the identity transformation. It's easy to see that p-functors $\mathsf{Id}_{\_}$ are units of the sequential composition. To ensure associativity we need to consider p-functors up to an equivalence of their parameter spaces. Two parallel p-functors $f\colon \mathbf{A} \xrightarrow{\mathbf{P}} \mathbf{B}$ and $\hat{f}\colon \mathbf{A} \xrightarrow{\hat{\mathbf{P}}} \mathbf{B}$, are *equivalent* if there is an isomorphism $\alpha\colon \mathbf{P} \to \hat{\mathbf{P}}$ such that two parallel functors $f\colon \mathbf{P} \to [\mathbf{A}, \mathbf{B}]$ and $\alpha; \hat{f}\colon \mathbf{P} \to [\mathbf{A}, \mathbf{B}]$ are naturally isomorphic; then we write $f \approx_\alpha \hat{f}$. It's easy to see that if $f \approx_\alpha \hat{f}\colon \mathbf{A} \to \mathbf{B}$ and $g \approx_\beta \hat{g}\colon \mathbf{B} \to \mathbf{C}$, then $f; g \approx_{\alpha\times\beta} \hat{f}; \hat{g}\colon \mathbf{A} \to \mathbf{C}$, i.e., sequential composition is stable under equivalence. Below we will identify p-functors and their equivalence classes. Using a natural isomorphism $(\mathbf{P}\times\mathbf{Q})\times\mathbf{R} \cong \mathbf{P}\times(\mathbf{Q}\times\mathbf{R})$, strict associativity of the functor composition and strict associativity of the Godement product, we conclude that sequential composition of (equivalence classes of) p-functors is strictly associative. Hence, ***pCat*** is a category.

Our next goal is to supply it with a monoidal structure. We borrow the latter from the sm-category $(\boldsymbol{Cat},\times)$, whose tensor is given by the product. There is an identical on objects embedding $(\boldsymbol{Cat},\times) \rightarrowtail \boldsymbol{pCat}$ that maps a functor $f\colon \mathbf{A} \to \mathbf{B}$ to a p-functor $\bar{f}\colon \mathbf{A} \xrightarrow{\mathbf{1}} \mathbf{B}$ whose parameter space is the singleton category $\mathbf{1}$. Moreover, as this embedding is a functor, the coherence equations for the associators and unitors that hold in $(\boldsymbol{Cat},\times)$ hold in ***pCat*** as well (this proof idea is borrowed from [17]). In this way, ***pCat*** becomes an sm-category. In a similar way, we define the sm-category ***pSet*** of small sets and parametrized functions between them — the codiscrete version of ***pCat***. The diagram in Fig. 7 shows how these categories are related.

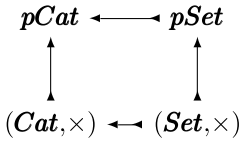

Fig. 7

### A.2 Ala-lenses as categorification of ML-learners

Figure 8 shows a discrete two-dimensional plane with each axis having three points: a space is a singleton, a set, a category encoded by coordinates 0,1,2 resp. Each of the points $x_{ij}$ is then the location of a corresponding sm-category of

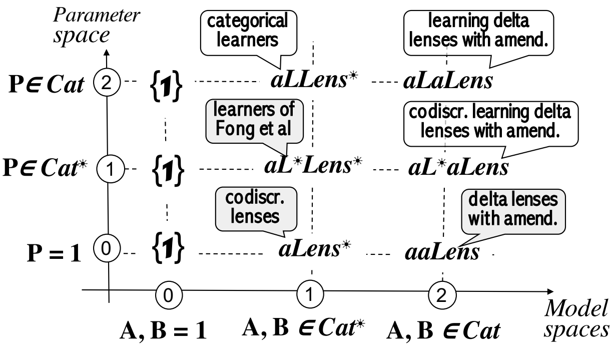

Fig. 8: The universe of categories of learning delta lenses

(asymmetric) learning (delta) lenses. Category $\{\boldsymbol{1}\}$ is a terminal category whose only arrow is the identity lens $\boldsymbol{1} = (\mathsf{id}_{\mathbf{1}}, \mathsf{id}_{\mathbf{1}})\colon \mathbf{1} \to \mathbf{1}$ propagating from a terminal category $\mathbf{1}$ to itself. Label $*$ refers to the codiscrete specialization of the construct being labelled: $\boldsymbol{L}^*$ means codiscrete learning (i.e., the parameter space $\mathbf{P}$ is a set considered as a codiscrete category) and $\boldsymbol{aLens}^*$ refers to codiscrete model spaces. The category of learners defined in [17] is located at point (1,1), and the category of learning delta lenses with amendments defined in the present paper is located at (2,2). There are also two semi-categorificated species of learning lenses: categorical learners at point (1,2) and codiscretely learning delta lenses at (2,1), which are special cases of ala-lenses.
